# Increased cardiorespiratory synchronization evoked by a breath controller based on heartbeat detection

**DOI:** 10.1186/s12938-019-0683-9

**Published:** 2019-05-20

**Authors:** Yumiao Ren, Jianbao Zhang

**Affiliations:** 1 Key Laboratory of Biomedical Information Engineering of Education Ministry, Xi’an Jiaotong University, Xianning West Road, Xi’an, 710049 China; 2 School of Electronics and Information Engineering, Xi’an Technological University, Xi’an, 710032 China

**Keywords:** Heart, Respiration, Cardiopulmonary phase synchronization, Voluntary cardiorespiratory synchronization

## Abstract

**Background:**

The cardiovascular and respiratory systems are functionally related to each other, but the underlying physiologic mechanism of cardiorespiratory coupling (CRC) is unclear. Cardiopulmonary phase synchronization is a form of cardiorespiratory coupling. However, it is difficult to study in experimental data which are very often inherently nonstationary and thus contain only quasiperiodic oscillations. So how to enhance cardiopulmonary synchronization and quantify cardiopulmonary synchronization, the changes in cardiac function under the conditions of cardiopulmonary synchronization, and the physiological mechanisms behind them are the main issues to be discussed in this paper.

**Results:**

The results showed that the cardiorespiratory synchronization significantly increased when breathing was controlled by heartbeat detection (*p* < 0.001). And the respiratory sinus arrhythmia (RSA) obviously decreased (*p* < 0.01) in the 2/2 mode and increased (*p* < 0.001) in the 4/4 mode. During the 2/2 breathing pattern compared with spontaneous breathing, systolic blood pressure (SBP) decreased (*p* < 0.05), and diastolic blood pressure (DBP), mean arterial blood pressure (MBP), and SV decreased significantly (*p* < 0.01). During the 4/4 breathing pattern compared to 2/2 breathing patterns, DBP, MBP, and cardiac output (CO) increased (*p* < 0.05), and stroke volume (SV) increased significantly (*p* < 0.01). When analyzing the relationships among these parameters, the RSA was found to be associated with the respiration rate in all respiratory patterns.

**Conclusions:**

We demonstrated that voluntary cardiorespiratory synchronization (VCRS) can effectively enhance cardiopulmonary phase synchronization, but cardiopulmonary phase synchronization and RSA represent different aspects of the cardiorespiratory interaction. It is found that cardiac function parameters such as the blood pressure and output per stroke could be affected by the number of heartbeats contained in the exhalation and inspiratory phase regulated through VCRS. So we can study cardiopulmonary phase synchronization by VCRS. It can be used to study in experimental data for the physiological mechanism of cardiopulmonary coupling.

## Background

It is well known that the heart and lung control systems are coupled with each other. Cardiac and respiratory rhythms in humans are synchronized. However, the underlying physiologic mechanism of cardiopulmonary synchronization is unclear. Research on cardiopulmonary synchronization has a certain guiding significance for the diagnosis and treatment of some diseases and for healthcare. Studies have shown that the cardiopulmonary synchronization of athletes and swimmers is superior to that of ordinary people [1, 2], and cardiopulmonary synchronization during the practice meditation is enhanced compare to that during natural breathing [3, 4]. These results seem to suggest that cardiopulmonary enhancement represents a better physiological state. So how to enhance cardiopulmonary synchronization and quantify cardiopulmonary synchronization, the changes in cardiac function under the conditions of cardiopulmonary synchronization, and the physiological mechanisms behind them are the main issues to be discussed in this paper.

The interaction between the cardiac and the respiratory systems is traditionally identified through the respiratory sinus arrhythmia (RSA), which accounts for the periodic variation of the heart rate within a breathing cycle. With the development of nonlinear dynamics, phase-synchronization analysis technology was used to analyze the relationships in cardiorespiratory coupling [1, 5–8]. More recently, phase synchronization between heartbeat and breathing has been studied using the synchrogram method [9], which first applied to check the different synchronous states and conversion rates of cardiorespiratory coupling by Schafer C [2]. Hoyer D improved the detection method of phase synchronization [10]. At present, the cardiorespiratory synchrogram method has been widely used in cardiopulmonary coupling research [11–13].

In this paper, we investigated the physiological mechanism of cardiopulmonary coupling based on enhancing cardiopulmonary synchronicity using the heartbeat to control breath. The only method of controlling respiratory-induced variation before 1964 was for subjects to hold their breath. In 1964, Schmitt was first put forward controlling breathing using synchronization with an electrocardiogram/vector cardiogram (ECG/VCG), which was called voluntary cardiorespiratory synchronization (VCRS) [14]. VCRS has been applied to study the changes in heart rate variability with human age and body position [15], to investigate respiratory effects on stroke volume [16], and to study the influence of respiration on changes in blood pressure and heart rate [17]. However, the enhancement of cardiopulmonary synchrony by VCRS has not been quantified and verified, and it is not discussed whether cardiopulmonary synchronization time is the influencing factor of cardiac function. These are important to further reveal the cardiopulmonary coupling mechanism.

Our aim is to use VCRS to study respiratory effects on blood pressure, heart rate, stroke volume, and cardiac output, using the phase-synchronization technique to quantify cardiopulmonary synchronization time. On the basis of synchronicity enhancement, we analyzed the effects of cardiopulmonary synchronicity on heart function and discussed the underlying physiologic mechanism of cardiorespiratory coupling.

## Methods

### Subjects

Thirty healthy male subjects (19–27 years old) voluntarily participated in the study. Each subject was given a medical survey questionnaire to ensure that they had no respiratory and cardiovascular diseases. The investigation was performed with the approval of the Xi’an Jiaotong University Ethics Committee, and all subjects signed an approved informed consent after the study procedures had been explained.

### Experimental protocol

The experiment was performed in a quiet laboratory between 8 p.m. and 10 p.m. and the temperature was controlled to 22–24 °C. The subjects were asked to avoid alcohol, tea, coffee, and strenuous exercises for 12 h and to get enough sleep the night before the experiment.

**Fig. 1:**
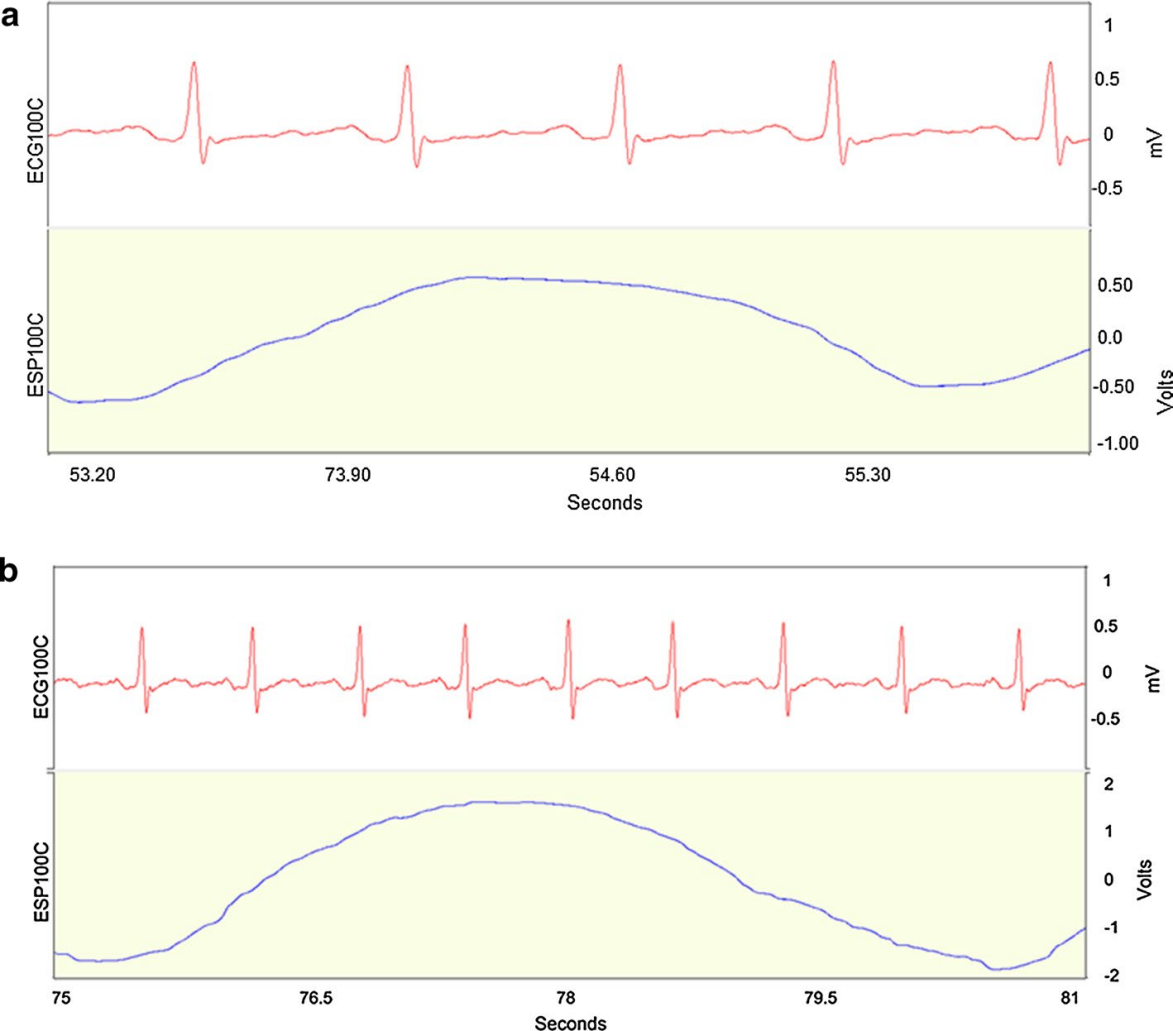
The relationship between ECG and respiration signals of a subject using the 2/2 and 4/4 breathing patterns. Above is the ECG signal, and below is breathing signal. **a** ECG and respiration signals of a subject in the voluntary cardiorespiratory synchronization 2*/*2 mode, during which the subject inspired for two heartbeats and expired for two heartbeats in one respiration cycle. **b** ECG and respiration signals of a subject in the voluntary cardiorespiratory synchronization 4*/*4 mode, during which the subject inspired for four heartbeats and expired for four heartbeats. The top trace shows the ECG; the bottom trace shows respiration measured by a respiration belt

Participants were seated comfortably. Before measurements were taken, participants rested and remained calm for 10 min. Then, the subjects received instructions for breathing in the sitting position. Three sets of data were tested with a rest period of 10 min between each set of measurements. The first measurement was made with subjects breathing spontaneously for 6 min. The subjects then exercised for 2 to 4 min to breath pace the VCRS, and the researchers made sure there were no problems during the procedure. The researcher was asked to follow the paced breathing VCRS mode for 6 min using a sound pattern from the ECG. This breath controller was developed in our laboratory. It outputs a sound signal by detecting the heartbeat signal and counting the heartbeats. The subject was instructed by the sound to breathe, synchronized with his/her heartbeat. The device signaled the subject when to inhale and exhale based on a fixed number of heart beats for each phase of the respiratory cycle: for example, inspire for the first two heartbeats and expire for the next two heartbeats in a 2/2 pattern. The 2/2 pattern was selected as the second measurement mode for 6 min. Figure 1a shows the ECG and respiration signals of a subject using this 2/2 breathing pattern. Another measurement mode was the 4/4 paced breathing VCRS pattern for 6 min. Figure 1b shows an example of this 4/4 breathing pattern. The 2/2 and 4/4 breathing patterns were chosen to increase and decrease the respiration rate, respectively, but still be subjectively comfortable for the participants.

### Physiological measurements

Respiration, ECG, and thoracic electric bioimpedance (TEB) signals were acquired online using a multichannel physiology recorder (MP150, Biopac, USA) and software (AcqKnowledge 4.2, Biopac systems) on a PC (Gateway) and collected at 1000 samples per second. Beat-to-beat arterial blood pressure was logged continuously via a noninvasive finger photoplethysmography (FMS, Finapres Measurement Systems, Arnhem, Netherlands). The synchronization method between the two instruments involves attaching the output interface (BNC) of the FMS to an analog input channel of the MP150.

The ECG was measured using disposable self-adhesive Ag/AgCl ECG electrodes, which were placed on the subject’s right arm, left leg, and right leg. The connection method was a standard bipolar ECG lead II that the left leg was connected to during the in-phase input of the amplifier, with the right arm connected to reverse-phase input, and the right leg linked to ground wire.

The TEB was recorded using NICO100C (Biopac system, Inc.). The NICO100C noninvasive cardiac output amplifier records the parameters associated with cardiac output measurements. It incorporates a precision high-frequency current source, which injects a very small (400 μA) measurement current through the thoracic volume, which is defined by the placement of a set of current source electrodes.

Respiration was recorded with a respiratory effort transducer strain assembly belt TSD201 (Biopac Inc.) that measures thoracic expansion and contraction. Voluntary cardiorespiratory synchronized breathing (VCRS) was controlled with the device that generated a sound to instruct the subject when to inhale and exhale, which was introduced above.

### Data analysis

#### Phase synchronization and synchrogram

Phase synchronization is a kind of cooperative performance between two weakly interacted oscillators. It is classically understood as phase locking and defined as [18]:

1 $$\varphi_{n,m} = \left| {n\varPhi_{1} - m\varPhi_{2} - \delta } \right| < {\text{const}},$$

where $n$ and $m$ are integers, $\varPhi_{1}$ and $\varPhi_{2}$ are phases of the two oscillators. The $n:m$ phase locking manifests as a variation of $\varphi_{n,m}$ around a horizontal plateau.

The synchrogram is a visualization tool which enables the detection of synchronization epochs in bivariate data. The synchrogram is constructed by plotting the corresponding normalized respiratory phase $\psi_{m} (t_{k} )$ for every heart beat within *m* respiratory cycles [19]:

2 $$\psi_{m} (t_{k} ) = \frac{1}{2\pi }\left( {\varPhi_{r} \left( {t_{k} } \right)\bmod 2m\pi } \right),$$

where $t_{k}$ is the time of the $k$ th R peak and $\varPhi_{r}$ is the corresponding respiratory phase.

The respiratory phase, $\varPhi_{r} \left( {t_{k} } \right)$, is calculated by the method based on the marker events as the following formula:

3 $$\varPhi_{r} \left( {t_{k} } \right) = 2\pi \frac{{t - t_{k} }}{{t_{k + 1} - t_{k} }} + 2\pi k,\quad t_{k} \le t < t_{k + 1},$$

where $t_{k}$ is the time of the onset of $k$ th expiration. The local maximum of respiratory signal is taken for the marker events of respiratory oscillator. In a perfect $m:n$ phase locking, $\psi_{m} \left( {t_{k} } \right)$ exactly attains the same $n$ different values within $m$ adjacent respiratory cycles, and the synchrogram consists of $n$ horizontal strips.

The method of phase recurrences based on a heuristic approach was used to quantify the cardiorespiratory synchrogram. Detection with phase recurrences offered the best temporal resolution and the highest number of synchronized sequence [20]. Generally, a $n:m$ synchronization will be identified if the difference between the normalized relative respiratory phase corresponding to the $\left( {i + n} \right)$ th R peak and the one corresponding to the $i$ th R peak is within a defined tolerance ε. This condition has to be fulfilled for at least $k$ successive R peaks:

4 $$\begin{aligned} & \exists k > 1,\quad \left| {\psi_{\text{m}} \left( {t_{i + n} } \right) - \psi_{\text{m}} \left( {t_{n} } \right)} \right| < \varepsilon , \\ & \quad i \in \left\{ {l, \ldots ,\;l + k - 1, \;0 \le l \le N_{r} - k + 1} \right\} \\ \end{aligned},$$

where $N_{r}$ is the total number of R peaks. To be compatible with the description of parallel horizontal lines during synchronization, $k \ge m$ needs to be fulfilled. This procedure allows a detection of a structure of parallel horizontal lines with a length of 2 m successive normalized relative phases.

It is most valuable in cases where one of the signals resembles a point process. The synchrogram is a stroboscopic view of the phase of the respiration signal at the times of R waves. R peaks were detected using the combination of wavelet transforms and thresholding methods [21]. The peaks and troughs at breathing signal were extracted using the algorithm of threshold value. The times of R peaks at ECG and inspiratory onsets at respiratory signal were obtained and served as event markers for synchrogram, as shown in Fig. 3. The duration of the synchronization epochs is calculated, which is used as the index of synchronization strength in the article.

#### Respiratory sinus arrhythmia (RSA)

Respiratory Sinus Arrhythmia is used to explore the connection between respiration and changes to heart rate. This RSA index is computed using the peak-valley method [22]. This method uses both a recorded ECG Lead II signal and a respiration signal. The respiration signal is used to locate periods of inhalation and exhalation. Inhalation begins at valleys in the signal while expiration at peaks. RSA index is the average difference between the highest and lowest heart rate (HR) during each respiratory cycle (HR Max − HR Min) [23], expressed in milliseconds.

#### Arterial blood pressure

**Fig. 2:**
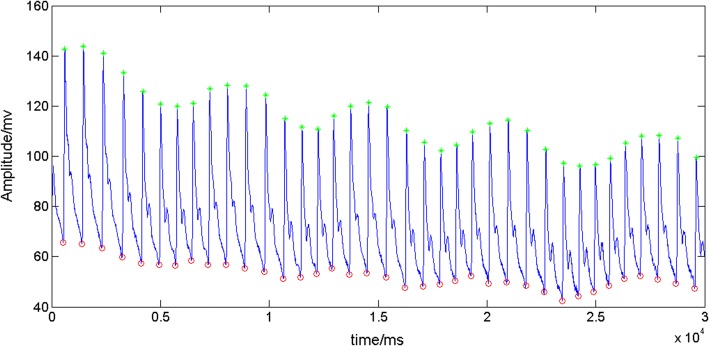
The peaks and troughs in the blood pressure signals of a subject. Systolic blood pressure (SBP) calculated by the peak is marked with cross symbols. Diastolic blood pressure (DBP) calculated by the trough is marked with circle

The peaks and troughs of the blood pressure signal were extracted for systolic and diastolic blood pressures by combining them with the ECG signal. The mean arterial blood pressure (MAP) (= (2 × diastolic)/3 + systolic/3) was calculated from systolic and diastolic blood pressures. The method of extracting feature points in the blood pressure signal incorporated into the ECG signal is an advantage of synchronous collection of blood pressure and ECG signals. The maximum and minimum of the blood pressure signal matched the cardiac cycle between two adjacent R wave peaks. Each cardiac cycle includes one systolic blood pressure and one diastolic blood pressure. The algorithm has a high detection rate, as shown in Fig. 2.

#### Stroke volume and cardiac output

The stroke volume (SV) and cardiac output (CO) can be derived from the popular noninvasive method of impedance cardiography. CO is defined as the volume of blood pumped each minute ($= {\text{SV}} \times {\text{HR}}$). SV can be calculated from the Kubicek formula [16]:

5 $${\text{SV}} = \rho \frac{{L^{2} }}{{Z_{0}^{2} }}\left( {\frac{{{\text{d}}Z}}{{{\text{d}}t}}} \right)_{\text{max} } \times {\text{LVET}},$$

where ${\text{SV}}$ is stroke volume, i.e., volume of blood pumped by left ventricle in a single beat (mL beat), $\rho$ is blood resistivity at 100 kHz (typical value for a normal hematocrit = 150 Ω cm), *L* is mean distance between inner pair of electrodes (cm), $Z_{0}$ is the average basal thoracic impedance (Ω), LVET is left ventricular ejection time(s), $\left( {{\text{d}}Z/{\text{d}}t} \right)_{\text{max} }$ is the maximum value of (${\text{d}}Z/{\text{d}}t$) signal (Ω s^−1^), and (${\text{d}}Z/{\text{d}}t$) is the derivative of cardiac systolic impedance.

### Statistical analysis

Statistical analyses were performed with SigmaPlot software (Systat Software, Inc. USA). If the data were normally distributed, paired t tests were used. Otherwise, rank sum tests were used. A *p* value < 0.05 was statistically considered significant, and data were represented as the mean ± SEM. Pearson’s correlation coefficients were used for the correlation analyses.

## Results

### Changes in the cardiorespiratory synchronization time (Syn), RSA, and breath rate (BR)

Compared with spontaneous breathing, Syn was significantly increased (*p* < 0.001 for the 2/2 breathing pattern, *p* < 0.01 for the 4/4 breathing pattern). RSA obviously decreased (*p* < 0.01) in the 2/2 mode and increased (*p* < 0.001) in the 4/4 mode. In contrast, the BR was significantly increased (*p* < 0.001) during the 2/2 breathing pattern, and it was significantly decreased (*p* < 0.001) during the 4/4 breathing pattern (Table 1).

**Table 1:** Syn and BR during spontaneous breathing and 2/2 and 4/4 breathing patterns

	Spt	2/2	4/4
Syn (s)	19.79 ± 3.59	84.43 ± 10.77***	60.85 ± 14.39**
RSA (ms)	76.84 ± 6.91	56.10 ± 6.38**	137.97 ± 11.71***^###^
BR (min)	14.82 ± 0.62	18.73 ± 0.59***	9.51 ± 0.25***^###^

Data are presented as the mean ± SEM

Spt, spontaneous breathing; 2/2, 2/2 breathing pattern; 4/4, 4/4 breathing pattern; Syn, synchronization time; RSA, HR Max minus HR Min; BR, breath rate

** and *** *p* < 0.01 and *p* < 0.001, respectively, vs the spontaneous breathing pattern (paired t test). ^###^ *p* < 0.001, respectively, for a comparison of the = 4/4 breathing pattern and the 2/2 breathing pattern

**Fig. 3:**
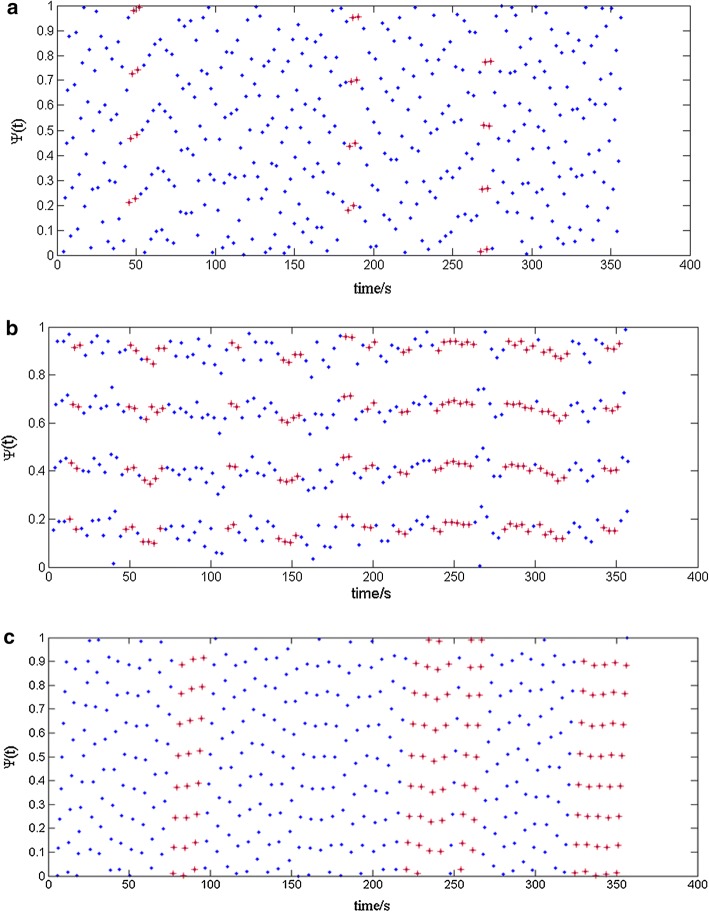
Typical cardiorespiratory synchrogram of one subject. Typical cardiorespiratory synchrograms of one subject for spontaneous breathing (**a**), the 2/2 breathing pattern (**b**), and the 4/4 breathing pattern (**c**). Synchronization, marked with cross symbols, is characterized by the arrangement of the wrapped phase in horizontal lines. In this subject, 4:1 phase synchronization was detected for both 2/2 breathing pattern and spontaneous breathing, but the synchronization epoch in 2/2 breathing pattern obviously longer. 8:1 phase synchronization was detected for the 4/4 breathing pattern

The results showed that cardiorespiratory synchronization was significantly increased by breath control based on heartbeat detection. A typical cardiorespiratory synchrogram of one subject is shown in Fig. 3. During spontaneous breathing, synchronization was found in 26 subjects, and 4 subjects did not exhibit any synchronization. The average synchronization epoch was 19.79 s in 360 s, which was 5.50% of the total recordings. During the 2/2 breathing pattern, synchronization was found in 29 subjects, and 1 subject did not exhibit any synchronization. The mean synchronization epoch was 84.43 s in 360 s, which indicated that heartbeat and breath rate were synchronized in 23.45% of the total recording; this result was far longer than that of spontaneous breathing. During the 4/4 breathing pattern, the average synchronization epoch in all 24 subjects was 60.85 s in 360 s, which was 16.90% of the total recording; this result was far longer than that of spontaneous breathing. Furthermore, the breath rate was in accordance with the breathing controller based on heartbeat detection. These results suggest that the levels of cardiorespiratory coupling were significantly higher in the 2/2 and 4/4 breathing modes than during spontaneous breathing.

### Effect on blood pressure, HR, SV, and CO

To determine the effect of breath triggered by ECG enhancing cardiopulmonary synchronicity on the cardiovascular system, we analyzed cardiovascular function parameters during the 2/2 and 4/4 breathing patterns relative to those during spontaneous breathing pattern and between the 2/2 and 4/4 breathing patterns (Table 2). These cardiovascular function parameters include blood pressure (SBP, DBP, and MBP), heart rate (HR), stroke volume (SV), and cardiac output (CO). During the 2/2 breathing pattern compared with spontaneous breathing, SBP decreased (*p* < 0.05), and DBP, MBP, and SV decreased significantly (*p* < 0.01). During the 4/4 breathing pattern compared with spontaneous breathing, all of the parameters showed no obvious change. During the 4/4 breathing pattern compared to the 2/2 breathing pattern, SBP, DBP, MBP, and CO increased (*p* < 0.05), and SV increased significantly (*p* < 0.01); no obvious difference was found in SBP and HR.

**Table 2:** Cardiac function parameters during spontaneous breathing and the 2/2 and 4/4 breathing patterns

	Spt	2/2	4/4
SBP (mmHg)	114.81 ± 2.57	112.10 ± 2.74*	114.81 ± 2.69^#^
DBP (mmHg)	61.47 ± 1.65	59.29 ± 1.83**	61.52 ± 1.79^#^
MBP (mmHg)	79.25 ± 1.84	76.89 ± 2.03**	79.28 ± 1.97^#^
HR (beat/min)	73.04 ± 2.10	74.66 ± 2.36	74.05 ± 2.11
SV (mL/beat)	144.22 ± 5.66	137.61 ± 5.77*	145.65 ± 5.53^##^
CO (L/min)	10.40 ± 0.42	10.12 ± 0.43	10.56 ± 0.42^#^

Data are presented as the mean ± SEM

Spt, spontaneous breathing; 2/2, 2/2 breathing pattern; 4/4, 4/4 breathing pattern; SBP, systemic blood pressure; DBP, diastolic blood pressure; MBP, mean blood pressure; HR, heart rate; SV, stroke volume; CO, cardiac output

* and ** *p* < 0.05 and *p* < 0.01, respectively, vs spontaneous breathing (paired *t* test). ^#^ and ^##^ indicate *p* < 0.05 and *p* < 0.01, respectively, for comparison of the 4/4 breathing pattern and the 2/2 breathing pattern

### Correlation among Syn, RSA, BP, and breath rate

**Fig. 4:**
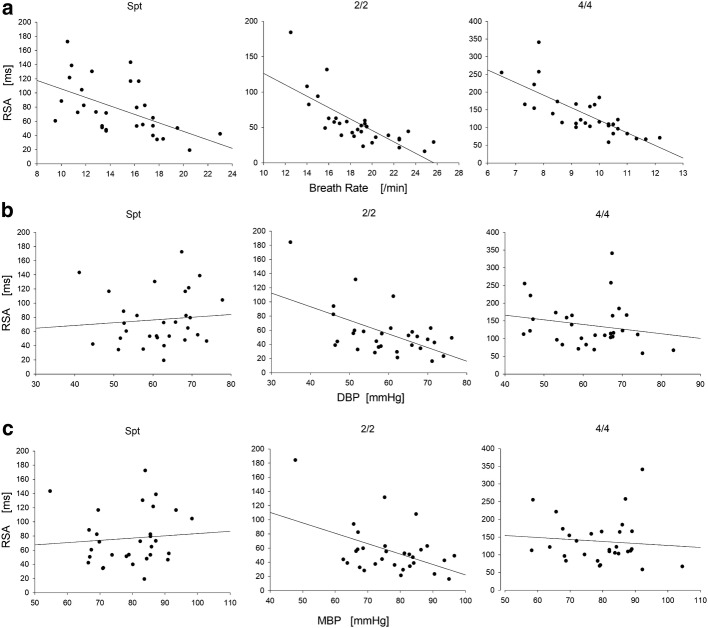
Correlation analysis of the power spectral energy of HRV and breath rate. Scatterplots for RSA and respiratory rate during spontaneous breathing (Spt), the 2/2 breathing pattern (2/2), and the 4/4 breathing pattern (4/4) (**a**). Scatterplots for RSA and DBP during spontaneous breathing (Spt), the 2/2 breathing pattern (2/2), and the 4/4 breathing pattern (4/4) (**b**). Scatterplots for RSA and MBP during spontaneous breathing and the 2/2 and 4/4 breathing modes (**c**)

To study the mechanism of cardiopulmonary coupling, we analyzed the correlation coefficients among the cardiopulmonary synchronization time and blood pressure, cardiac output, stroke output, and respiration rate. No significant correlation was found. When analyzing the correlation between RSA and these parameters, RSA was found to be associated with the respiration rate and BP in the 2/2 mode (Fig. 4). RSA was significantly negatively correlated with the respiration rate in all the respiratory mode (Spt, *r* = − 0.535, *p* = 0.002; 2/2 mode, *r* = − 0.741, *p* = 0.000003; *r* = − 0.757, *p* = 0.000001). RSA was obviously negatively correlated with DBP and MBP in the 2/2 respiratory mode (*r* = − 0.551, *p* = 0.002; *r* = − 0.468, *p* = 0.009). No obvious correlation was found in spontaneous breathing and 4/4 breath pattern.

## Discussion

Phase synchronization can be used to analyze cardiopulmonary coupling [1, 2, 11]. We analyzed the synchronization time of the heart and respiration in 2/2, 4/4, and spontaneous breathing patterns by using phase synchronous graphs and found that controlling respiration by heartbeat can significantly enhance cardiopulmonary coupling and control cardiopulmonary synchronization. Compared with spontaneous breathing, the synchronous time of the heart rate and respiration in 2/2 and 4/4 modes was significantly enhanced, the respiratory rate increased significantly during the 2/2 pattern, and the respiratory rate decreased significantly during the 4/4 pattern (Table 1). In addition, compared to the 2/2 breathing mode, the breathing rate was significantly reduced in the 4/4 breathing mode, but the synchronization time did not change significantly. These results show that the synchronization time is inconsistent with the change in respiration rate, which is agreement with Ref. [24]. So the enhancement of synchronization time is not caused by the change of respiratory rate. At the same time, the method of adjusting phase synchronization by VCRS is to control the initial phase and the ratio of heartbeat to respiratory cycle through heartbeat, which is important for enhancing phase-synchronization time [11]. This showed that VCRS can effectively enhance cardiopulmonary synchronization and control the number of heartbeats contained in the exhalation and inspiratory phase of the respiratory cycle. It provides experimental conditions for studying the physiological mechanism of cardiopulmonary coupling.

In this paper, we used VCRS to study the circulatory system and found that blood pressure and stroke volume were reduced in the 2/2 mode by VCRS. Through analysis, we think that this is related to the number of heartbeats contained in the exhalation and inspiratory phase regulated by VCRS. Compared with spontaneous breathing, the 2/2 respiratory pattern resulted in decreased blood pressure and stroke volume (Table 2), and there were no significant changes in mean HR because vagal tone did not change [23]. During the 2/2 respiratory pattern, the ratio of inhalation time to exhalation time of the respiratory cycle was approximately 1:1, and both the inhalation and exhalation phases contained two ECG cycles. In spontaneous breathing mode, the inhalation phase contained two ECG cycles, and the exhalation phase contained three ECG cycles [25], so the ratio of inhalation time to exhalation time was approximately 1:1.5. Compared with spontaneous breathing, the duration of inhalation increased in the whole respiratory cycle in the 2/2 breath mode. When inhaling, the decrease in pleural pressure resulted in increased right ventricular filling, which decreased left ventricular filling and stroke volume and further reduced systolic blood pressure. Exhalation has the opposite effect and was accompanied by a delayed effect of the increased right ventricular stroke volume due to inhalation. The maximum left ventricular stroke volume occurred during the posterior half of the exhalation phase. When the breathing amplitude increased, namely, the amount of the moisture increased, the magnitude of change in pleural pressure increased, and the effect became more pronounced [26, 27]. Therefore, the blood pressure and stroke output decreased in the 2/2 mode relative to those during spontaneous respiration. The maximum left ventricular stroke volume occurred during the latter half of the exhalation phase, although the time ratio of the inhalation phase to the exhalation phase was 1:1 in the 4/4 mode, the exhalation time had 4 ECG cycles. The exhalation time was sufficient compared to that of the 2/2 mode and showed little difference compared to that of spontaneous breath. Therefore, in the 4/4 mode, blood pressure, stroke, and cardiac output increased compared to the 2/2 mode, and there was no significant change compared to spontaneous breath. It indicates that blood pressure and output per stroke could affected by changes in chest pressure. In our study, it showed that the decrease in blood pressure and output per stroke is related to the mode by VCRS.

Our results demonstrate that cardiopulmonary phase synchronization and the traditionally studied respiratory sinus arrhythmia represent different aspects of the cardiorespiratory interaction. This is consent to previous studies [13, 24, 28]. In this study, the strength of phase synchronization is represented by phase-synchronization time, and the strength of RSA is represented by HR Max − HR Min. We investigated the relationship between phase synchronization and RSA. Our analyses did not reveal a statistical relation between the degree of cardiopulmonary phase synchronization and the strength of RSA. RSA was affected by respiration rate (Fig. 4a). However, cardiopulmonary phase-synchronization time was not correlated with respiration rate. And the changes of cardiopulmonary phase-synchronization time are different with RSA in all breath patterns (Table 1). These differences are due to the fact that, whereas RSA is a measure of the amplitude of variation of the heartbeat intervals within the breathing cycles, phase synchronization is characterized by the clustering of heartbeats at specific phases in the breathing cycle. This clustering is independent of the amplitude of heart rate modulation [24]. Cardiorespiratory phase synchronization is a type of cardiorespiratory coupling that manifests through a predilection for heart beats to occur at specific points relative to the phase of the respiratory cycle [29], which can be controlled by VCRS. However, the RSA is quite obviously another manifestation of the cardiorespiratory interaction [30]. In our study, RSA was significantly changed in all respiration modes, which was correlated with breath frequency and BP (Fig. 4). However, it is related to blood pressure only in 2/2 breathing mode. It is probable that RSA is mainly affected by breath frequency. Cardiopulmonary phase synchronization and RSA are different forms of the cardiorespiratory interaction, which can be cooperatively or independently used in cardiopulmonary coupling studies.

## Conclusions

Our study demonstrates that VCRS can effectively enhance cardiopulmonary phase synchronization, although phase synchronization is difficult to study in experimental data which are very often inherently nonstationary and thus contain only quasiperiodic oscillations. We can study cardiopulmonary phase synchronization by VCRS. In our experimental data, we found that 2/2 mode can lower blood pressure, and we think that this is related to the number of heartbeats contained in the exhalation and inspiratory phase regulated by VCRS. It may be possible to control the cardiovascular parameters by controlling respiratory rate and the number of heartbeats contained in the exhalation and inspiratory phase of the respiratory cycle through VCRS, thereby regulating cardiac function.

## Data Availability

The datasets used during the current study are available from the corresponding author on reasonable request.

## Abbreviations

CRC: cardiorespiratory coupling
RSA: respiratory sinus arrhythmia
SBP: systolic blood pressure
DBP: diastolic blood pressure
MBP: mean arterial blood pressure
CO: cardiac output
SV: stroke volume
VCRS: voluntary cardiorespiratory synchronization
ECG/VCG: electrocardiogram/vector cardiogram
HR: heart rate
LVET: left ventricular ejection time
Syn: cardiorespiratory synchronization time
BR: breath rate
Spt: spontaneous breathing
BP: blood pressure
